# Rapid Weight Loss and Dietary Inadequacies among Martial Arts Practitioners from Poland

**DOI:** 10.3390/ijerph15112476

**Published:** 2018-11-06

**Authors:** Anna Anyżewska, Igor Dzierżanowski, Agnieszka Woźniak, Magdalena Leonkiewicz, Agata Wawrzyniak

**Affiliations:** 1 Department of Human Nutrition, Faculty of Human Nutrition and Consumer Sciences, Warsaw University of Life Sciences, Nowoursynowska 159c, 02-776 Warsaw, Poland; info@dietetyk-sportowy.pl (I.D.); agnieszka_wozniak1@sggw.pl (A.W.); magdalena.leonkiewicz@gmail.com (M.L.); agata_wawrzyniak@sggw.pl (A.W.); 2 Laboratory of Food and Nutrition Hygiene, Military Institute of Hygiene and Epidemiology, Kozielska 4, 01-163 Warsaw, Poland

**Keywords:** nutrition, body weight, combat sports, athletes

## Abstract

Healthy nutrition and maintaining a normal body weight are very important for reducing the risk of various diseases not only among the general population, but also among athletes, especially combat sport athletes. The aim of the study was to evaluate the prevalence of rapid weight loss (RWL) and to indicate eventual dietary inadequacies among professional martial arts practitioners. Sixty-two male athletes (aged: 23 ± 4) completed a questionnaire (i.e., frequency of food group consumption, questions about training and RWL) and single 24-h dietary recall. This study confirmed the high prevalence of RWL in athletes (58%) for two to three days before the competition, which allowed for reduction of 3.4 ± 1.0 kg (4.3 ± 1.5%) of their body weight. Many dietary inaccuracies were found such as: lower than recommended by nutrition experts, level of consumption of dairy products, grain products, fruit, and vegetables, and insufficient intake of energy, carbohydrates, minerals (especially iodine, potassium, calcium) and vitamins (especially D, folate, C, E) during the training day. Adequate nutrition is a natural support for the whole training process, and may allow for regulation of body weight in a longer period and in a safer manner; thus, there is a need for nutrition education dedicated to athletes and their trainers.

## 1. Introduction

The process of training, as well as genetic factors, adequate nutrition, and hydration are crucial for achieving the best results in sports. A well-balanced diet should meet the athlete’s energy, macro- and micro-nutrient requirements, and may significantly influence his or her success, because it affects not only the exercise capacity, but also supports the recovery, thus optimizing the whole training process [1,2]. Academy of Nutrition and Dietetics, Dietitians of Canada, and the American College of Sports Medicine (ACSM) provided guideline to promote optimal health and performance across different scenarios of training and competitive sport, including the appropriate type, amount, and timing of intake of food, fluids, and supplements [2]. Detailed recommendations for carbohydrate, protein, fat and micronutrients were provided. All general dietary recommendations should be fine-tuned with individual consideration especially of total energy needs, specific training needs. Therefore, athletes should be referred to a registered dietitian for a well-chosen nutrition strategy and personalized nutrition plan [2].

In sports where weight classes are determined (e.g., combat sports), athletes often present peculiar attitudes to nutrition questions [3,4]. In combat sports, usually each athlete must measure his weight on the competition day and cannot exceed his weight class [3]. They often introduce unhealthy eating practices, such as skipping meals or restricting selected products or groups of products from the diet. Alternate overeating and fasting syndrome, periodical use of laxatives or diuretics are also often observed. This may lead to a depletion of essential nutrients in their diets. Adequate nutrition that meets the individual needs of an athlete would allow maintenance of a healthy body weight throughout the whole season, without the use of methods for rapid weight loss (RWL). Rapid weight loss is an intentional total body mass reduction used by athletes a few days before the start of competition. However, research shows that combat sport athletes instead of maintaining adequate nutrition during the preseason phase, often decide to use RWL methods before competitions, reducing over 5% of their body weight within a few days [5,6,7]. Inappropriate and excessive weight loss techniques (e.g., saunas, use of plastic or rubber suits, severe dieting, vomiting, laxatives, and diuretics, etc.) are extremely dangerous and should be or already are (e.g., diuretics) prohibited.

There are limited studies that concurrently assess RWL and nutrition practices among combat sport athletes, in Poland and in the world [8,9]. Research carried out in athletes indicated the widespread use of RWL a few days before competitions, and diet irregularities in diets such as insufficient daily intake of energy, fluids, and carbohydrates, as well as fiber, calcium, vitamin C and folate [6,7,8,9,10,11,12,13]. Such behaviors may compromise the physical performance and cause symptoms such as: headaches dizziness or nausea. Moreover, Morales et al. [14] demonstrated negative effects of rapid weight loss prior to competition on perceptual motor skill performance in judo athletes. Therefore, we conducted a study, which aim was to evaluate the prevalence of RWL and to indicate eventual dietary inadequacies among professional combat male athletes in Poland.

## 2. Materials and Methods

The study was conducted in a period from January to May 2014 year among 62 males who trained in combat sports professionally. Participants were recruited from professional sport clubs in Poland, using the snowball method [15]. Inclusion criteria of the participants in the study were as follows: (1) martial art training experience—at least 3 years; (2) at least one participation in the competition of nationwide rank; (3) regularity and minimum 4.5 h per week of martial art training; (4) lack of chronic diseases. Athletes had to meet all the criteria prior to the research, approval of the ethics committee at the Institute of Food and Nutrition in Warsaw (date: 2 October 2012) and informed consent from each participant was received.

### 2.1. Rapid Weight Loss

The athletes completed a questionnaire containing questions about age, weight, and height (to calculate the BMI [(kg/m^2^; self-reported)). BMI is not appropriate for athletes because of the excess weight from muscle mass, but it is used when there are no fat mass measures. Detailed information on the number of training (for all sport activities) hours per week, the training experience and the kind of combat sport trained was gathered. All participants trained martial arts at least 4.5 h per week. Questions about the use of RWL prior to the competitions, the length of RWL periods and the number of kilograms lost were also included in the questionnaire. For this study, authors defined RWL as an intentional total body mass reduction used by athletes two to three days before the start of competition.

### 2.2. Dietary Assessment

#### 2.2.1. Food Frequency Questionnaire

The athletes completed Food Frequency Questionnaire, that included questions about the frequency of consumption (never, occasionally, 1–2 times per week, 3–6 times per week, daily) of ten selected food groups (dairy, groats, rice, pasta, vegetables, fruits, fish, nuts, chocolate, bars, cakes, cookies, salty snacks, alcohol) in the previous three months. The Food Frequency Questionnaire was developed on the basis of the validated for polish population Food Frequency Questionnaire [16] and in accordance with the Methodical guide for testing the diet, developed by The Committee of Human Nutrition, Polish Academy of Sciences [17]. The questionnaire contained additional questions about using dietary and sports supplements in the previous three months.

#### 2.2.2. 24-h Dietary Recall

Single 24-h dietary recall was conducted by dietitians to determine the nutrition irregularities on the training day, and the diet during that day was typical for each of the athlete. The portion sizes of consumed foods were assessed using the photo album of products and dishes [18] and data were calculated with the software based on tables of nutritional values of foods and dishes [19]. Calculating the total intake of energy and other nutrients, all consumed foods, dishes, drinks, and supplements as well as processing losses and plate waste [20] were taken into account. For each participant, the intake of nutrients was compared with the dietary recommendations [21], EAR (estimated average requirement) or AI (adequate intake), and recommendations of the International Society of Sports Nutrition [2]. In this study, only salt derived from food products was considered. The percentage of energy from protein, carbohydrates, and fat was referred to the recommendations for Polish population [21].

### 2.3. Statistical Analysis

The Statistica PL v.10.0 computer program (StatSoft. Inc., Tulsa, OK, USA) was used for all statistical analysis. Because the values were not normally distributed (according to the Shapiro–Wilk test), the Spearman correlation test was used and *p* values < 0.05 were considered statistically significant. Correlations between the number of training hours per week and nutrient intake, as well as between nutrient intake and food frequency consumption, were indicated.

## 3. Results

All athletes belonged to sport clubs in Poland and trained in judo (60%), kickboxing (15%), Brazilian ju-jitsu BJJ (11%), mixed martial arts MMA (11%), or boxing (3%). Athletes participated in the competition of nationwide rank at least once, and 39% of athletes belonged to the National Team. Participants were training on average 8.9 ± 3.0 h per week (of combat sports) and training experience was 11 ± 5 years (Table 1).

### 3.1. Rapid Weight Loss

Forty-two athletes (68%) declared the weight reduction before competitions, and 6% of participants did not decrease body mass because of starting in the “open” category, without any weight limits. Among the athletes who reduced body mass, 36 (86%) used the RWL methods, two to three days before a competition. So, it was 58% of surveyed athletes (36 of 62). Most of athletes used dehydration (69%), restricted diets (61%), increased exercise intensity (39%), and thermogenics (17%). Athletes could reduce 1.6–7.2% (on average 4.3 ± 1.5, median: 4.3) of body mass (1.5–5.0 kg; on average 3.4 ± 1.0, median: 3.0) due to used rapid weight loss methods.

### 3.2. Dietary Assessment

#### 3.2.1. Food Frequency Questionnaire

**Figure 1:**
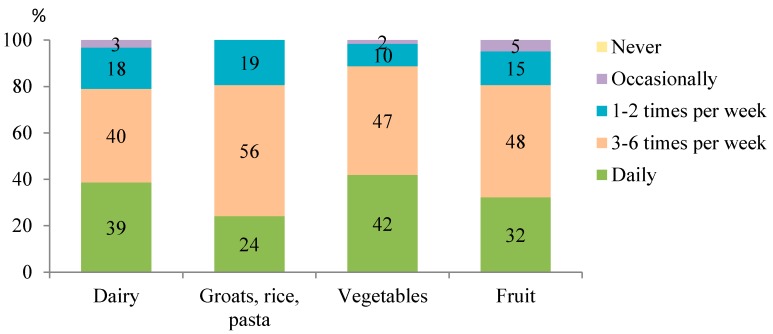
The most often consumed food groups (*n* = 62).

**Figure 2:**
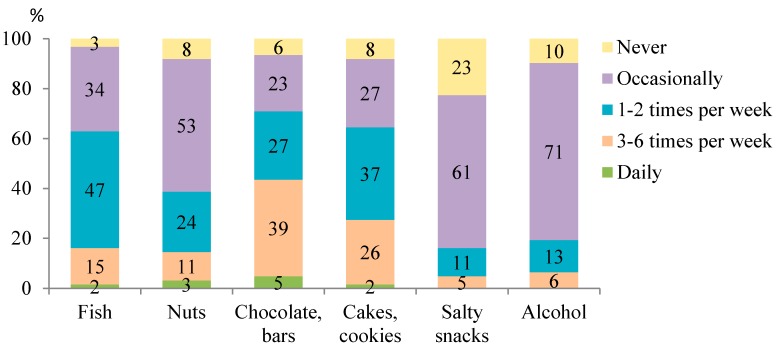
The least often consumed food groups (*n* = 62).

The food groups that were most often consumed daily were vegetables (by 42% athletes), milk and dairy products (39%), fruits (32%), and grain products such as groats, rice, and pasta (24%) (Figure 1). Half of the athletes consumed fish once or twice a week, and occasionally nuts (Figure 2). In contrast, the least often (i.e., occasionally or not at all) consumed foods were salty snacks (by 84% athletes) and alcohol (by 81%). Approximately two-thirds of athletes ate chocolate, chocolate bars, cakes, and cookies a few times a week.

#### 3.2.2. 24-h Dietary Recall

Energy intake during a training day was lower than recommended level in almost all athletes and the mean value equaled 2377 ± 645 kcal (Table 2). Athletes consumed on average 5.0 ± 1.3 L of water from foods and drinks. The percentage of energy from protein was too high (23 ± 7%), but protein intake was adequate in almost all athletes when it was calculated per kg of body weight, and equaled on average 1.6 ± 0.5 g/kg b.w. However, 17% of respondents had too high intake of protein—over 2 g/kg b.w. The mean consumption of carbohydrates was 3.6 ± 1.1 g/kg b.w. and of fats—0.9 ± 0.4 g/kg b.w. The percentage of the energy from those macronutrients equaled 50 ± 9% and 28 ± 9%, respectively. Although the mean percentage of energy from carbohydrates was adequate, only 10% of athletes provided carbohydrates in the range recommended by the International Society of Sports Nutrition (5–7 g/kg b.w.) [2]. Statistical analysis showed a positive correlation between the number of training hours per week and the intake of energy (r = 0.30; *p* = 0.017), protein (r = 0.30; *p* = 0.017), carbohydrates (r = 0.30; *p* = 0.020), and fluids (r = 0.27; *p* = 0.037), indicating that athletes who trained more, also consumed larger amounts of above-mentioned dietary components and energy.

Based on the 24 h recall assessment the quantities of minerals and vitamins provided with foods, liquids, and supplements during the training day were not appropriate, i.e., lower than recommended level. Only 50% of participants consumed adequate amounts of potassium, and the recommendations for calcium and magnesium were met in only 45% and 68% of athletes, respectively. Most of the athletes met the recommendations for iron (97%) and zinc (84%), and all met recommendations for phosphorus. The intake of B-vitamins, with the exception for folate, were adequate, on average in 81% of respondents. Antioxidant vitamins were consumed in insufficient amounts, and only 44%, 58%, and 71% of athletes met the recommendations for vitamins C, E, and A, respectively. The lowest percentage of participants who consumed the vitamins according to recommendations were found in the case of vitamin D (5%) and folate (35%). Statistical analysis indicated positive correlations between the intake of calcium and the frequency of dairy products consumption (r = 0.29; *p* = 0.021); vitamin A intake and the frequency of fish consumption (r = 0.46; *p* < 0.001), as well as between folate intake and the frequency of nuts consumption (r = 0.27; *p* = 0.034).

The majority of athletes (81%) declared the use of dietary or sports supplements. Among athletes, the most popular were isotonic drinks (82%), BCAA (46%), carbohydrate supplements (44%), vitamins and minerals (38%), and protein supplements (32%). Less often used supplements were creatine (20%), other amino acids (18%), stimulants with caffeine (18%), omega-3 supplements (8%), weight gainers (6%), thermogenic (6%), and beta-alanine (2%). In addition, two-thirds of those athletes said that taking the supplements was their own decision, and only 8% received such recommendations from a dietician.

## 4. Discussion

This study confirmed the high prevalence of RWL in combat sports athletes for two to three days before the competition and many dietary and nutritional inadequacies.

### 4.1. Rapid Weight Loss

This study confirmed the common use of RWL methods (58%) a few days before a competition among the Polish combat sport athletes. It was found that the athletes could reduce 7% of body weight in two to three days before a competition. Rapid weight loss was also observed among martial arts practitioners in other studies [5,6,9,22,23] (Table 3). Some of the other studies reported use of RWL in longer time periods—e.g., about seven days before competitions (6.22). Thus, the fact of such huge differences in body weight within such a short time is even more worrying because it can be harmful to health [5]. Rapid weight loss methods, such as taking laxatives, diuretics, use of plastic or rubber suits, and sauna are harmful to performance and health. Moreover, RWL affects physical and cognitive capacities, and may increase the risk of death. Extremely alarming is that the weight reduction practices often start at very early ages [8] Moreover, the use of intensive trainings and restrictive diets, are commonly accompanied with using saunas, wearing plastic or rubber suits, or limiting liquid intake [6,22,23,24]. In addition, RWL is not always associated with a reduction of body fat. Fleming et al. [8] underlined that, in lean taekwondo athletes, the amount of adipose tissue had not changed during a weight reduction period, suggesting that the reduced body weight resulted from dehydration and loss of lean body mass. The key in the regulation of muscle mass is not only the amount of protein consumed during the day, but rather multifactorial interactions among protein sources, dose, and timing [25]. 

Most commonly used technique for RWL is dehydration, which does not necessarily involve a restriction of fluid intake [3]. Dehydration can be unintentional, e.g., as a consequence of negative energy balance, since water is essential in the process of degradation of protein and glycogen. Intended dehydration usually involves increased sweating that results from increased both the number and intensity of trainings [3]. According to Mendes et al. [26], athletes using RWL regularly are more exposed to its negative health consequences as there is no organism adaptation to the stress connected with RWL. Artioli et al. [27] proposed a few rules that are aimed at improving the techniques of weight reduction among judo athletes. The basis for a weight control program are as follow: (1) competition should begin within 1 h after weigh-in, at the latest; (2) each athlete is allowed to be weighed-in only once; (3) rapid weight loss as well as artificial rehydration (i.e., saline infusion) methods are prohibited during the entire competition day; (4) athletes should pass the hydration test to get their weigh-in validated; (5) an individual minimum competitive weight (male athletes competing at no less than 7% and females at no less than 12% of body fat) should be determined at the beginning of each season; (6) athletes are not allowed to compete in any weight class that requires weight reductions greater than 1.5% of body weight per week [27]. It is worth underlying that well balanced diet—i.e., the adequate intake of energy, macronutrients, and liquids—should be the only method that allows regulation of body weight for a longer period and in a healthier way. 

### 4.2. Dietary Inadequacies

Adequate nutrition is an essential element for each athlete in the whole training process. Diet should be properly balanced according to gender, age, type, and duration of physical activity during a day, as well as the training phase. The energy cost is associated with the type, intensity, and duration of physical activity [28]. The intensity in combat sports is very high, and the effort is more interval than uniform. According to Ainsworth et al. [29] the mean energy cost during the training in combat sports equals 10.3 MET (i.e., 10.3 kcal/1 kg b.w./1 h). In the present study and in others, many inaccuracies in the diets of combat sports athletes were indicated (Table 4) [8,11,30,31,32]. Extremely alarming is lower than recommended fruit and vegetable frequency consumption. Only 32% of martial arts practitioner consumed fruits every day and merely 42% of athletes consumed vegetables every day. Insufficient frequency of consumption of food groups such as dairy, grains, vegetables, and fruit were also found by Fleming et al. [8] and Ubeda et al. [30]. Insufficient intake of such products may lead to insufficient intake of such nutrients such as calcium, some B-vitamins, or antioxidant vitamins, what was demonstrated in this study. Those nutrients are essential for proper functioning of the organism, especially in the case of elevated physical activity [2]. In addition, lower than recommended level an intake of energy, sustained over a long time, can contribute to a reduction in exercise capacity, poorer recovery after exercise, and thus can lead following training to be less efficient. A survey carried out among taekwondo athletes in Korea indicated much higher energy intake than in our study, which equaled on average 3754 ± 307 kcal and 4657 ± 414 kcal during the summer and winter grouping, respectively [33]. Although many studies show various intakes of macronutrients among combat sport athletes [8,31,33,34], the majority indicate too low amounts of carbohydrates, similarly to our findings. Insufficient intake of carbohydrates during the training day, and especially after intense physical exercise, can reduce the exercise capability and delay recovery and glycogen replenishment. In our study as well as in others, protein intake was adequate in the vast majority of athletes and averaged 1.6 g/kg b.w. what exceeds the minimum of 1.2 g/kg b.w., needed to maintain and build muscle mass as well as improve regeneration of cells and tissues damaged during training [35]. Consumption of selected minerals and vitamins was not satisfactory, especially since the requirements for most of athletes can increase with high physical activity [2]. Many authors emphasize that inadequate calcium intake along with low energy intake has a deleterious effect on bones, what can be particularly dangerous for combat sports athletes [36,37,38]. Low intake of potassium, calcium, and magnesium can lead to athletes decreased levels in the body and, consequently, may weaken the muscles and ability of shortening, and also disturb the metabolic processes [1]. Extremely low vitamin D intake is alarming especially in the context that its skin synthesis among indoor athletes probably is insufficient throughout the year. Vitamin D is essential for bone health, immune function, inflammatory modulation, optimal muscle function, and performance and thus may affect sport performance [39]. There is a recommendation that athletes should have 25(OH)D level assessed annually [40].

The use of dietary supplements was quite common among the athletes in our study and was more various than in MMA fighters, who mainly took fish oil supplements (64%), thermogenic supplements (36%), beta-alanine (36%), and creatine (27%) [9]. In contrast, among judo athletes, the most popular were protein supplements, calcium, creatine, carbohydrates, and vitamins [41]. Also, in that study, majority of the athletes made their own decisions about the usage and type of supplements. It was found that dietary supplement use in the study group was very high (81%), and martial arts practitioners were more likely to use them than soldiers from the US Army [42]. Awareness and misconceptions of dietary supplements health risk among athletes might be a serious issue too.

Athletes are at risk of having insufficient amounts of crucial nutrients due to the improper food product selection and consumption, what was found in the present study. Lower than recommended intake of energy, micro-, and micronutrients as well as RWL may not only negatively affect exercise capacity and recovery but mainly the health of athletes.

## 5. Strengths and Limitations of the Study

There are limited numbers of studies that assess concurrently the nutrition and the use of RWL methods among combat sports athletes. Moreover, studies are conducted in small groups (e.g., in 7 taekwondo athletes) [8]. Besides, some research on prevalence RWL in combat sports does not take into account the amount of the reduced body weight expressed as a percentage of baseline weight, which hinders interpretation of the results. The present study included absolute as well as relative loss of weight, and was conducted in a big group of 62 athletes. In addition, researchers often apply the expression of RWL to periods of weight reductions that last 7 days [6,22], or even 60 days [26]. The authors of present study think that RWL is only related to a few days before the competitions, thus the period of two to three days before competitions was used. It turned out it was a valid assumption, because 86% of athletes that had reduced their weight, did so two to three days before the competition.

However, the limitations of this study are the lack of anthropometric measurements (i.e., skin-fold thickness, fat mass, total body water) and the use of respondents’ declarations. The authors agree that the study should be conducted over a longer time, that would monitor the eating habits and behaviors, during preparation phase, and in the last few days before the competition, and combined with anthropometric measurements. The other limitation of this study is the use of a 24-h dietary recall, because of the lack of intra-individual variability. That is why we also used food frequency consumption. However, according to the Magkos and Yannankolia [43], the use of a single 24-h dietary recall might be an alternative when there is no possibility of using the instrument more than once. There are other studies that use this method [44,45,46,47].

## 6. Conclusions

This study confirmed the high prevalence of RWL in combat sports athletes (58%) for two to three days before the competition, which allowed to reduce on average 3.4 kg (4.3%) of their body weight. Many dietary and nutritional inaccuracies were found such as: lower than recommended consumption of dairy products, grain products, fruit and vegetables, and also insufficient intake of energy, carbohydrates, minerals (especially iodine, potassium, calcium) and vitamins (especially D, folate, C, E) during the training day.

Adequate nutrition is a natural support for the whole training process, may allow regulation of body weight for a longer period and in a safer manner as well as enhance recovery, thus there is a need for a more detailed, thorough dietary analysis of long-term dietary and RWL behaviors and nutrition education dedicated to athletes and their trainers.

## Figures and Tables

**Table 1:** Characteristics of Study Participants (*n* = 62).

Variable	Mean	±	SD	Median	Min	Max
Age (years)	23	±	4	23	18	34
Weight (kg)	83.0	±	12.6	81.5	59.0	130.0
Height (cm)	180	±	6	180	168	194
Body-mass Index (kg/m2)	25.6	±	2.8	25.3	20.2	32.2
Training (martial arts) (h/week)	8.9	±	3.0	8.0	4.5	15.0

**Table 2:** Energy and Nutrient Intake on The Training Day (*n* = 62).

Component	Mean	±	SD	Median	IQR	Recommendations	Prevalence [%] of Sufficient Intake
Energy (kcal)	2377	±	645	2294	814	3136–5644	3
Water (L)	5.0	±	1.3	4.9	2.2	2.5 (AI)	100
Protein (g)	133	±	47	123	49	-	-
Protein (g/kg m.c.)	1.6	±	0.5	1.5	0.6	0.73 (EAR) 1.2–2.0 *	98 63
Protein (%E)	23	±	7	22	7	10–20	34
Carbohydrates (g)	298	±	95	297	122	-	-
Carbohydrates (g/kg m.c.)	3.6	±	1.1	3.5	1.6	5–7 *	10
Carbohydrates (%E)	50	±	9	50	11	45–65	71
Fiber (g)	23.3	±	8.2	21.8	10.2	25 (AI)	68
Fat (g)	73	±	33	68	49	-	-
Fat (g/kg m.c.)	0.9	±	0.4	0.8	0.4	-	-
Fat (%E)	28	±	9	29	10	20–35	71
Sodium (mg)	2044	±	995	1957	1384	1500 (AI)	66
Potassium (mg)	3575	±	964	3505	1412	3500 (AI)	50
Calcium (mg)	844	±	424	719	599	800 (AI)	45
Phosphorus (mg)	1949	±	532	1873	638	580 (EAR)	100
Magnesium (mg)	388.9	±	123.5	374.8	126	330 (EAR)	68
Iron (mg)	13.0	±	3.9	13.1	5.4	6 (EAR)	97
Zinc (mg)	12.8	±	3.7	12.9	5.4	9.4 (EAR)	84
Iodine (μg)	47.1	±	22.8	45.0	28.6	95 (EAR)	5
Vit. A (retinol equivalent) (μg)	1208	±	1504	774	972	630 (EAR)	71
Vit. D (μg)	3.9	±	5.0	2.2	2.6	15 (AI)	5
Vit. E (alpha-tocopherol equivalent) (mg)	10.7	±	5.0	10.5	5.6	10 (AI)	58
Thiamine (mg)	1.6	±	0.6	1.4	0.6	1.1 (EAR)	79
Riboflavin (mg)	2.2	±	0.9	2.1	1.2	1.1 (EAR)	90
Niacin (mg)	31.2	±	14.1	29.8	16.5	16 (EAR)	85
Vit. B_6_ (mg)	2.9	±	1.0	2.8	1.3	1.3 (EAR)	98
Vit. B_12_ (μg)	5.9	±	6.4	4.7	2.8	2.0 (EAR)	97
Folate (μg)	293	±	108	289	121	320 (EAR)	35
Vit. C (mg)	85.2	±	66.9	67.7	74.5	75 (EAR)	44

IQR—interquartile range, %E—percentages of total energy, * Thomas et al. [2].

**Table 3:** Rapid Weight Loss Prevalence and Magnitude in Combat Sports’ Athletes.

Martial Arts	Training Experience (Years)	Rapid Weight Loss				Reference
		Prevalence	Days before Competition	kg	% Body Mass	
Judo, kickboxing, BJJ, MMA, boxing	11 ± 5 *	58%	2–3	3.4 ± 1.0 3.0 ** (1.5–5.0) ***	4.3 ± 1.5 4.3 (1.6–7.2)	this study
Judo, jujitsu, karate, taekwondo	8 ± 5	63%	max 7	3.6 ± 1.5 (0.3–10.0)	5.3 ± 3.5	Brito et al., 2012 [6]
MMA	5 ± 5	-	2	5.8 ± 3.3 (0–10.0)	-	Bounty et al., 2012 [9]
Judo	9 ± 1	77%	max 7	4.5 ± 3.5 (1.0–10.0)	-	Fabrini et al., 2010 [22]
Judo	-	86%	7 ± 7 (1–60)	1.6 ± 1.6 (0.0–12.0)	2.5 ± 2.3 (0.0–16.0)	Artioli et al., 2010 [23]

BJJ—Brazilian jiu-jitsu, MMA—mixed martial arts; * mean ± standard deviation; ** median; *** range.

**Table 4:** Dietary Inadequacies among Professional Combat Male Athletes.

Martial Arts	Intake						Methods	Reference
	Selected Food Groups	Energy	Water	Macronutrients	Minerals	Vitamins		
Judo, kickboxing, BJJ, MMA, boxing (*n* = 62)	Vegetables and fruit, dairy, cereals - insufficient *	Insufficient	Sufficient	Carbohydrates - insufficient	Iodine, potassium, calcium - insufficient	Niacin, vit. D., folate, vit. C - insufficient	24-h dietary recall, FFQ	This study
Taekwondo (*n* = 7)	Vegetables and fruit, dairy - insufficient; highly processed foods - excessive	Insufficient	Insufficient	Carbohydrates-insufficient; fat - excessive	Calcium, zinc - insufficient	Vit. E - insufficient	Five-day food diary	Fleming et al., 2007 [8]
Taekwondo (*n* = 5)	-	Sufficient	Insufficient	Fiber - insufficient	Sufficient	Vit. A - insufficient	Three-day diet record	Rossi et al., 2009 [11]
Taekwondo, judo, boxing (*n* = 22)	Vegetables and fruit, cereals, eggs - insufficient	-	-	-	-	-	FFQ	Ubeda et al., 2010 [30]
Judo (*n* = 28)	-	Insufficient	Insufficient	Carbohydrates - insufficient	Potassium, calcium - insufficient	Sufficient	Five-day recall	Książek et al., 2014 [31]
Shorinji Kempo (*n* = 16)	-	Insufficient	-	-	Calcium - insufficient	Vit. D - insufficient	FFQ	Sumida et al., 2012 [32]

BJJ—Brazilian jiu-jitsu, MMA—mixed martial arts, FFQ—food frequency questionnaire; * pointed out by authors.
